# Supplementary material for: Accurate haplotype construction and detection of selection signatures enabled by high quality pig genome sequences
Source: Nat Commun. 2023 Aug 23;14:5126. doi: 10.1038/s41467-023-40434-3 (PMC10447580; doi:10.1038/s41467-023-40434-3)
Supplement: Supplementary file 1 — Supplementary Information [file 41467_2023_40434_MOESM1_ESM.pdf]

Supplementary Information

Accurate haplotype construction and detection of selection signatures enabled by high quality pig genome sequences

Authors: Xinkai Tong[1,2], Dong Chen[1], Jianchao Hu[1], Shiyao Lin[1], Ziqi Ling[1], Huashui Ai[1], Zhiyan Zhang[1*] & Lusheng Huang[1*]

[1]National Key Laboratory for Swine genetic improvement and production technology, Ministry of Science and Technology of China, Jiangxi Agricultural University, NanChang, Jiangxi Province, PR China.

[2]College of Life Sciences, Jiangxi Normal University, NanChang, Jiangxi Province, PR China.

[*]Corresponding authors: Prof. Dr. Zhiyan Zhang, Prof. Dr. Lusheng Huang

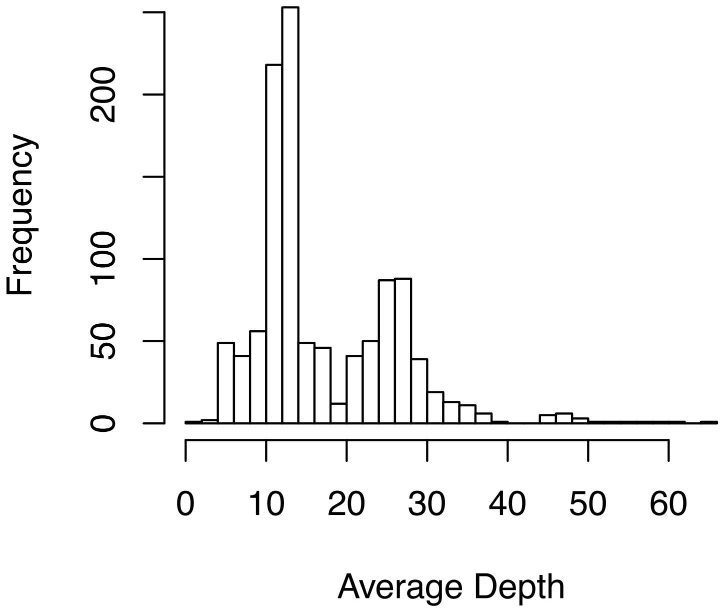

Supplementary Fig. 1 The frequency distribution of average sequencing depth of samples. A histogram is used to show the sequencing depth of samples. The x-axis denotes average sequencing depth (×) for each sample. The y-axis denotes the number of samples. Each bin is plotted as a bar whose height represents the sample size in that bin.

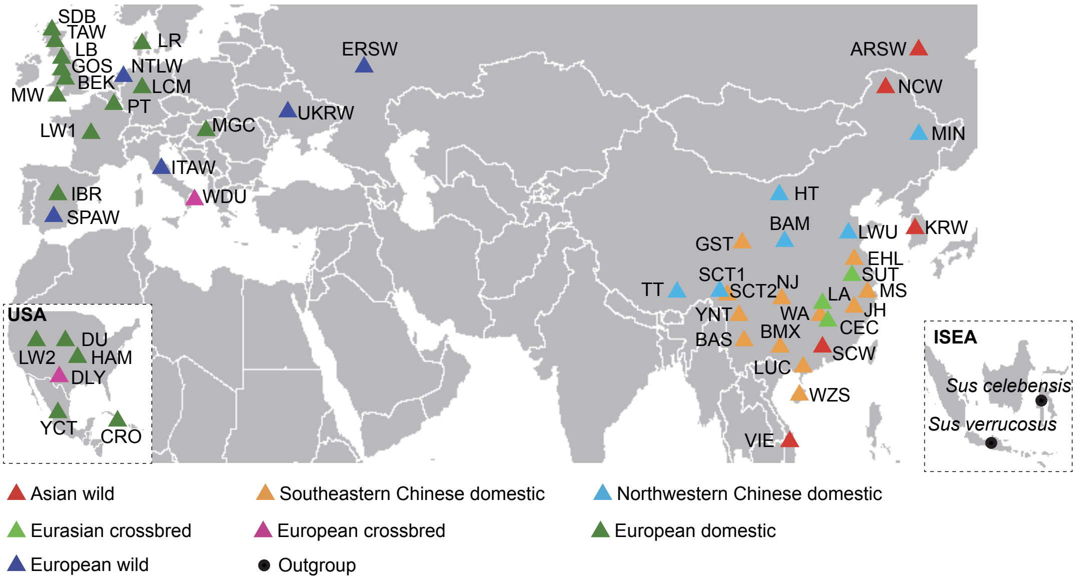

Supplementary Fig. 2 Geographic distribution (i.e., sampling sites or country of origin of breeds) of the samples analyzed in this study. Color codes of triangles correspond to the seven groups obtained in the phylogenetic tree. ISEA, islands of southeast Asia. ARSW, Asian Russia wild; ERSW, European Russia wild; BAM, Bamei; BAS, Baoshan; BEK, Berkshire; BMX, Bamaxiang; CRO, Creole; DU, Duroc; EHL, Erhualian; CEC or F1/F2/F3, Eurasian Crossbred; GOS, Gloucester old Spot; GST, Gansu Tibetan; HAM, Hampshire; HT, Hetao; IBR, Iberian pig; ITAW, Italian wild; JH, Jinhua; KRW, Korean wild; LA, Lean spotted pig; LB, Large Black; LCM, Leicoma; LR, Landrace; LUC, Luchuan; LW, LargeWhite; LWU, Laiwu; MGC, Mangalica; MS, Meishan; MW, Middle White; NCW, Northern chinese wild pig; NJ, Neijiang; NTLW, Netherland wild; PT, Pietrain; SCT, Sichuan Tibetan; SCW, Sourthern chinese wild; SDB, British Saddleback; SPAW, Spanish wild; SUT, Sutai; TAW, Tamworth; TT, Tibetan Tibetan; UKRW, Ukraine wild; VIE, Vietnam wild; WA, Wanan spotted; WDU, White Duroc; WZS, Wuzhishan; YCT, Yucatan; YNT, Yunnan Tibetan. The map is

generated through the ggplot2 package (https://ggplot2.tidyverse.org/reference/borders.html) in the R program. The ggplot2 package invokes the maps package to generate a world map (https://rdrr.io/cran/maps/man/world.html). This world map is imported from the public domain Natural Earth project (https://www.naturalearthdata.com).

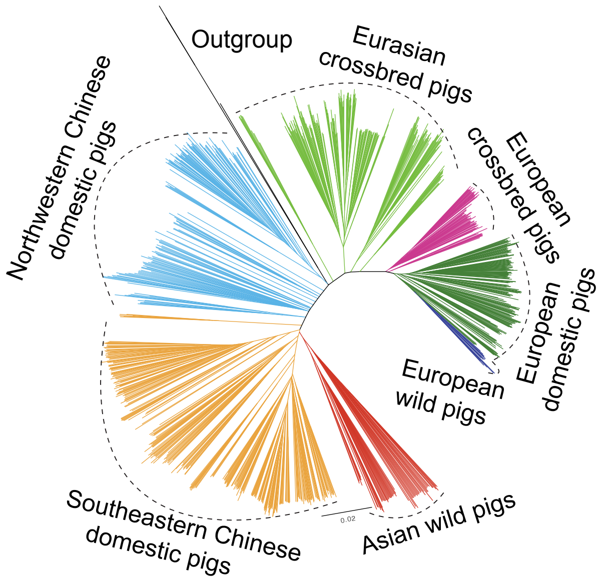

Supplementary Fig. 3 Neighbor-Joining phylogenetic tree, using *Sus verrucosus* (Javan warty pig) and *Sus celebensis* (Celebes warty pig) as outgroup. Each branch denotes an individual. All samples are used to construct the phylogenetic tree.

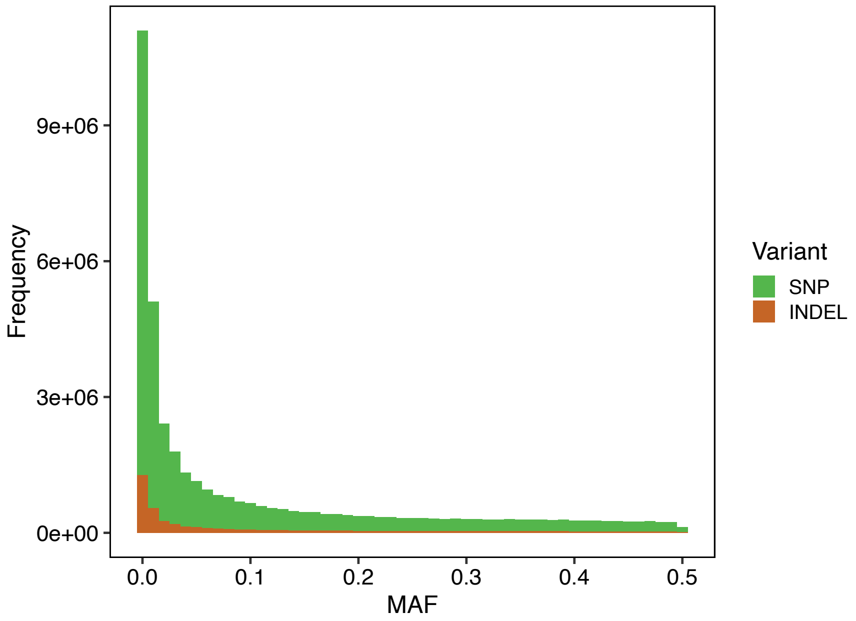

Supplementary Fig. 4 The minor allele frequency (MAF) distribution of SNPs and Indels. A histogram is used to show the frequency of variant under different MAF. The x-axis denotes minor allele frequency. The y-axis denotes the number of variants. Each bin is plotted as a bar whose height represents the number of variants in that bin.

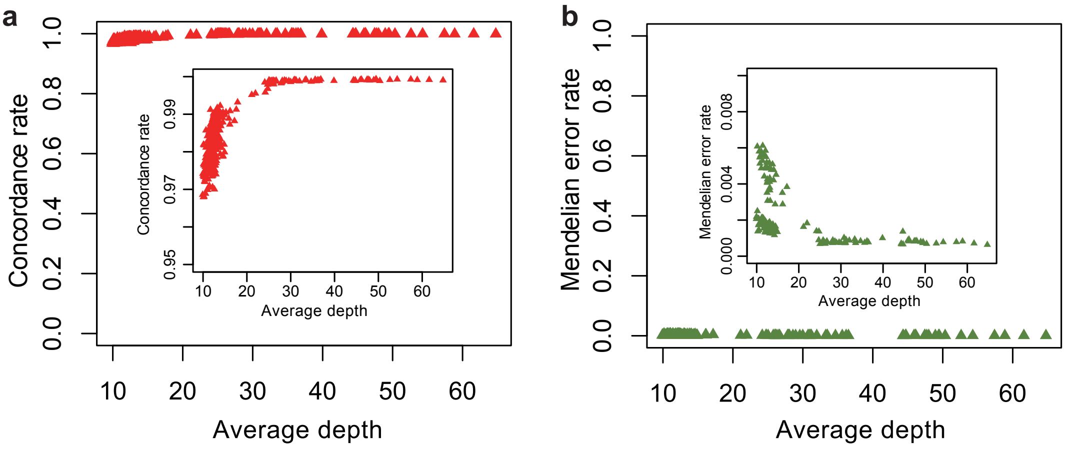

Supplementary Fig. 5 Validation the accuracy of variant genotyping. (a) The concordance rate between sequence genotypes and array genotypes. The x-axis shows average sequencing depth. **(b)** The Mendel Error rate estimated by summarizing error inherited variants in the 179 parent-offspring trios (44) or duos (135). Each triangle represents an individual.

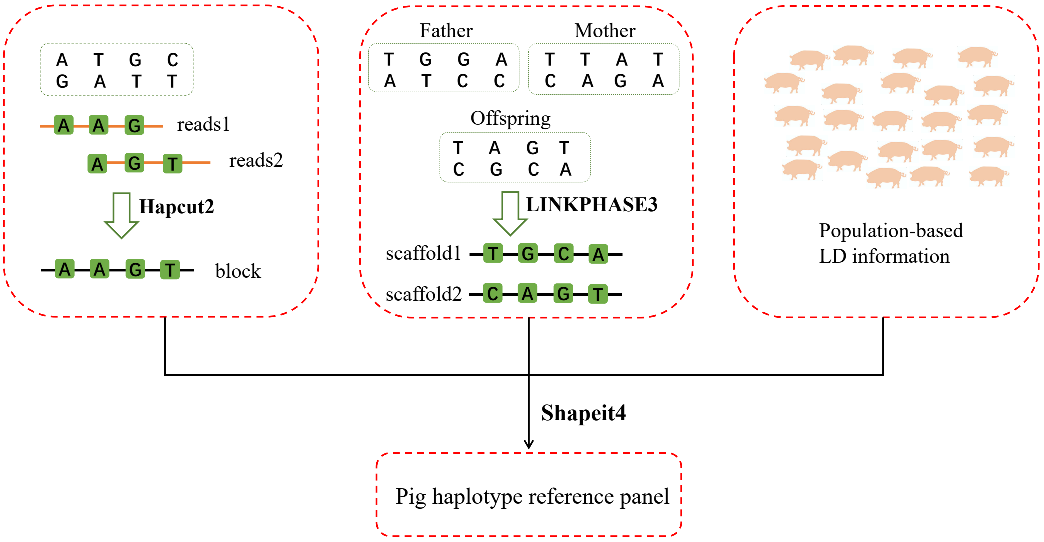

Supplementary Fig. 6 The schematic diagram for construction of the pig haplotype reference panel. The left, middle, right boxes represent haplotype blocks establishment, haplotype scaffolds build and Linkage Disequilibrium information extraction from population, respectively. Pig cartoons adapted from Chen, C., Zhou, Y., Fu, H. et al. Expanded catalog of microbial genes and metagenome-assembled genomes from the pig gut microbiome. Nat Commun 12, 1106 (2021). https://doi.org/10.1038/s41467-021-21295-0.

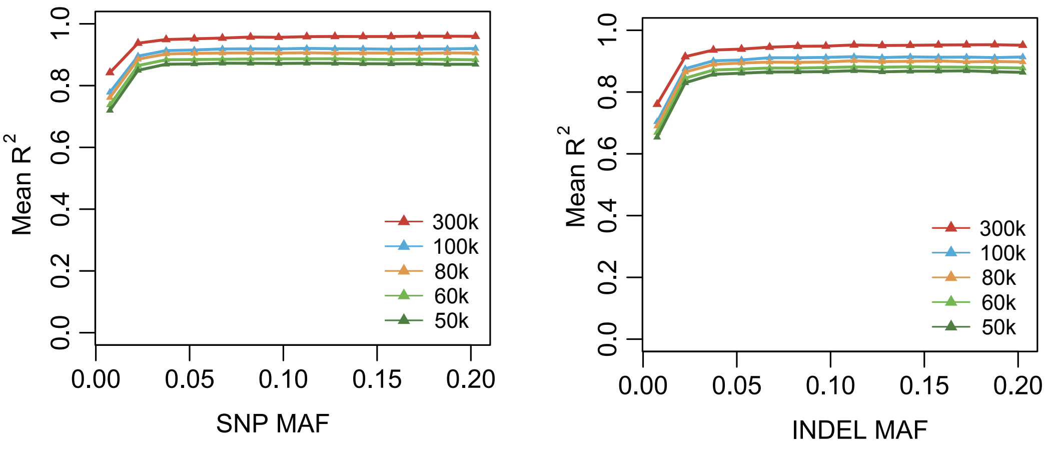

Supplementary Fig. 7 Imputation accuracy using ten common Chinese indigenous pigs (Erhualian, N = 4, Bamaxiang, N = 3, Laiwu, N = 3) as targets and the remaining 1854 haplotypes as reference panel. The x-axis shows the minor allele frequency of imputed variants. The y-axis shows imputation accuracy measured by average R^2 (squared Pearson correlation). The colored triangles represent the R^2 under different minor allele frequencies. The 50k, 60k, 80k, 100k, and 300k denote the number of randomly selected polymorphism sites being used for imputation.

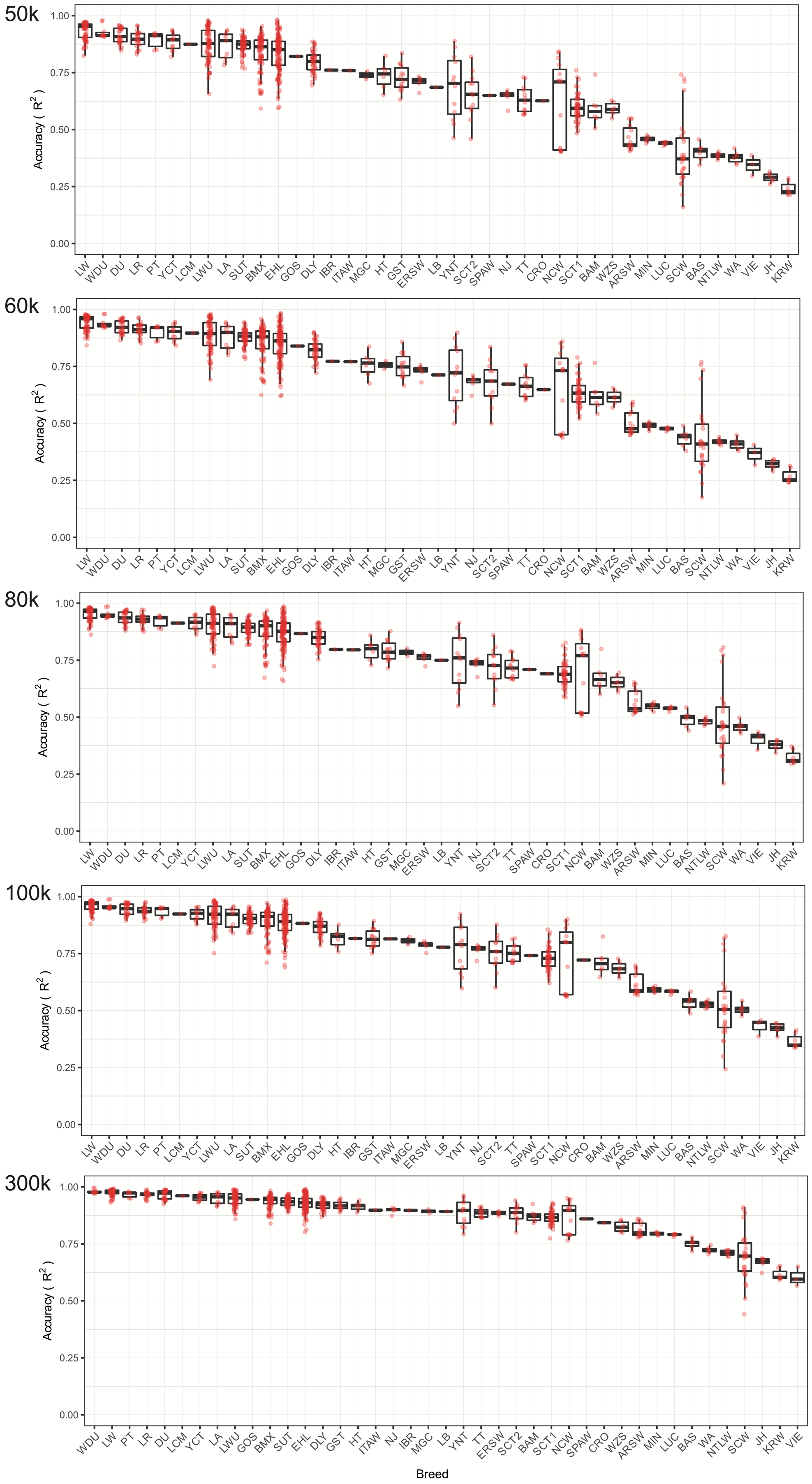
50k
60k
80k
100k
300k
Accuracy (R2)
1.00
0.75
0.50
0.25
0.00
LW WDU DU LR PT YCT LCM LWU LA SUT BMX EHL GOS DLY IBR ITAW MGC HT GST ERSW LB YNT SCT2 SPAW NJ TT CRO NCW SCT1 BAM WZS ARSW MIN LUC SCW BAS NTLW WA VIE JH KRW
LW WDU DU LR PT YCT LCM LWU LA SUT BMX EHL GOS DLY IBR ITAW HT MGC GST ERSW LB YNT NJ SCT2 SPAW TT CRO NCW SCT1 BAM WZS ARSW MIN LUC BAS SCW NTLW WA VIE JH KRW
LW WDU DU LR PT LCM YCT LWU LA SUT BMX EHL GOS DLY IBR ITAW HT GST MGC ERSW LB YNT NJ SCT2 TT SPAW CRO SCT1 NCW BAM WZS ARSW MIN LUC BAS NTLW SCW WA VIE JH KRW
LW WDU DU LR PT LCM YCT LWU LA SUT BMX EHL GOS DLY HT IBR GST ITAW MGC ERSW LB YNT NJ SCT2 TT SPAW SCT1 NCW CRO BAM WZS ARSW MIN LUC BAS NTLW SCW WA VIE JH KRW
WDU LW PT LR DU LCM YCT LA LWU GOS BMX SUT EHL DLY GST HT ITAW NJ IBR MGC LB YNT TT ERSW SCT2 BAM SCT1 NCW SPAW CRO WZS ARSW MIN LUC BAS WA NTLW SCW JH KRW VIE
Breed

Supplementary Fig. 8 Genotypes imputation accuracy for each breed included in the haplotype reference panel. The accuracy was evaluated as follows: 1) We sampled ten individuals as imputation targets and the remaining 1854 haplotypes as reference panel. The 50k, 60k, 80k, 100k, and 300k variants at autosomes were randomly selected to mimic chips. The genotypes of unselected variants were imputed for sampled ten individuals. 2) Step 1) was repeated until genotypes of all individuals were imputed once. 3) The imputation accuracy was measured by average R^2 (squared Pearson correlation) between sequenced genotypes and imputed genotypes. The letters on the upper left indicates the number of variants selected randomly for imputation. The y-axis represents imputation accuracy measured by R^2 (squared Pearson correlation). The x-axis denotes abbreviation of breeds. Each point represents an individual. Boxplots show median, 25th and 75th percentile, the whiskers indicate the minima and maxima, and the points laying outside the whiskers of boxplots represent the outliers. ARSW, Asian Russia wild; ERSW, European Russia wild; BAM, Bamei; BAS, Baoshan; BMX, Bamaxiang; CRO, Creole; DU, Duroc; EHL, Erhualian; GOS, Gloucester old Spot; GST, Gansu Tibetan; HT, Hetao; IBR, Iberian pig; ITAW, Italian wild; JH, Jinhua; KRW, Korean wild; LA, Lean spotted pig; LB, Large Black; LCM, Leicoma; LR, Landrace; LUC, Luchuan; LW, LargeWhite; LWU, Laiwu; MGC, Mangalica; NCW, Northern chinese wild pig; NJ, Neijiang; NTLW, Netherland wild; PT, Pietrain; SCT, Sichuan Tibetan; SCW, Sourthern chinese wild; SPAW, Spanish wild; SUT, Sutai; TT, Tibetan Tibetan; VIE, Vietnam wild; WA, Wanan spotted; WDU, White Duroc; WZS, Wuzhishan; YCT, Yucatan; YNT, Yunnan Tibetan.

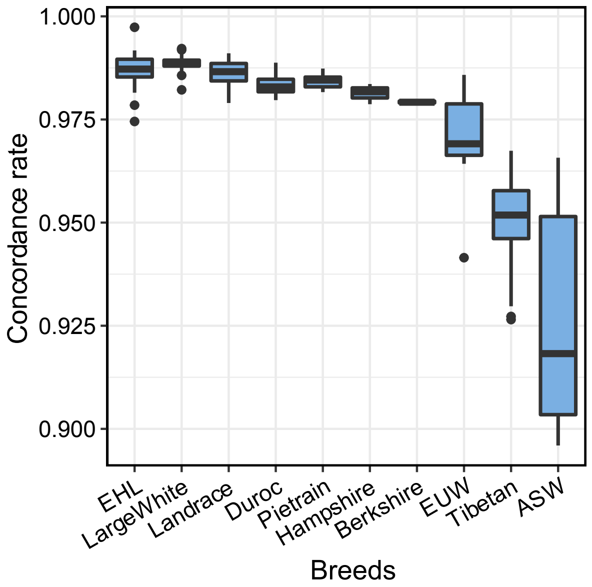

Supplementary Fig. 9 The concordance rate between sequencing genotypes and imputed genotypes for samples being removed for low call rate. Boxplots show median, 25th and 75th percentile, the whiskers indicate the minima and maxima, and the points laying outside the whiskers of boxplots represent the outliers. EHL (Erhualian), n = 22; LargeWhite, n = 20; Landrace, n = 12; Duroc, n = 4; Pietrain, n = 5; Hampshire, n = 3; Berkshire, n = 3; EUW (European wild pigs), n = 10; Tibetan, n = 31; ASW (Asian wild pigs), n = 10.

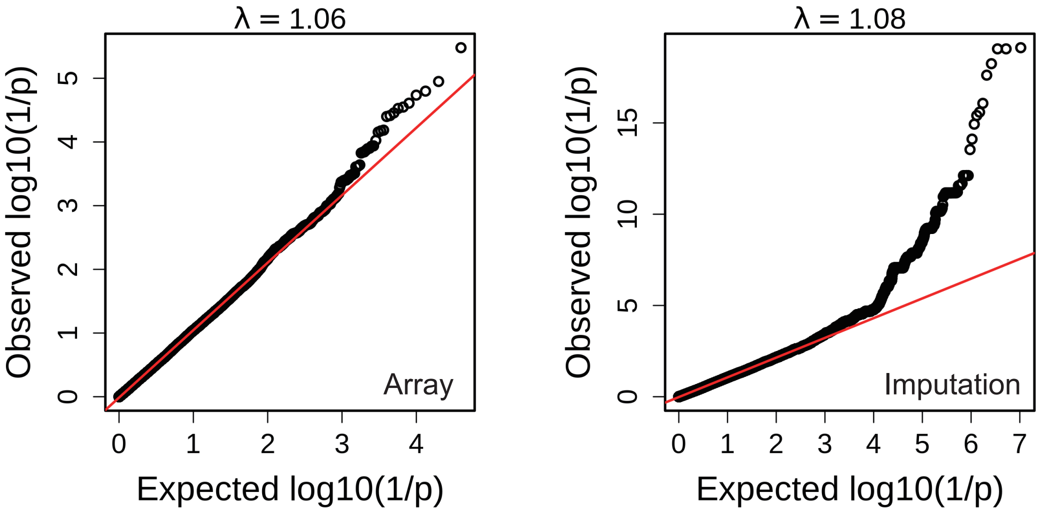

Supplementary Fig. 10 Quantile-quantile plots of array and imputation GWASs. Upper lambda denotes inflation factor of association test results. Each dot represents a variant. Red lines are fitted by a simple linear regression of function lm() implemented in R software.

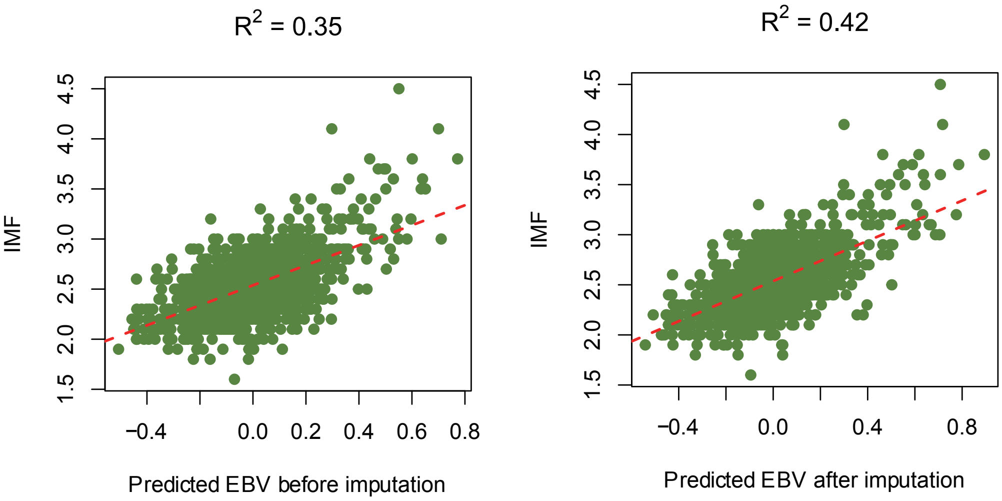

Supplementary Fig. 11 The phenotypic prediction ability of EBV before and after genotype imputation. Significant variants at the *P* < 0.01 level from GWAS were used for estimating the breeding value of IMF by cross-validated BLUP (leave-one-out). The x-axis shows the predicted EBV. The y-axis shows the phenotypic value of IMF. Each dot represents an individual. Pearson's correlation is employed to evaluate the phenotypic prediction ability of EBV, indicated by a R^2 (squared Pearson correlation) between phenotypic value and predicted EBV. The *P* values of correlation coefficient before and after imputation are 7.3×10^{-144} and 1.3×10^{-177}, respectively.

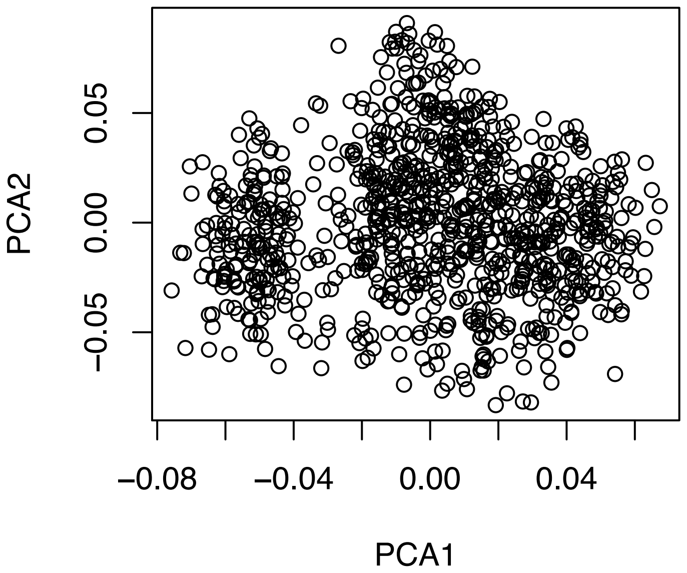

Supplementary Fig. 12 The principal component analysis using the first two principle components based on genotypes of F2 population. Each dot represents an individual.

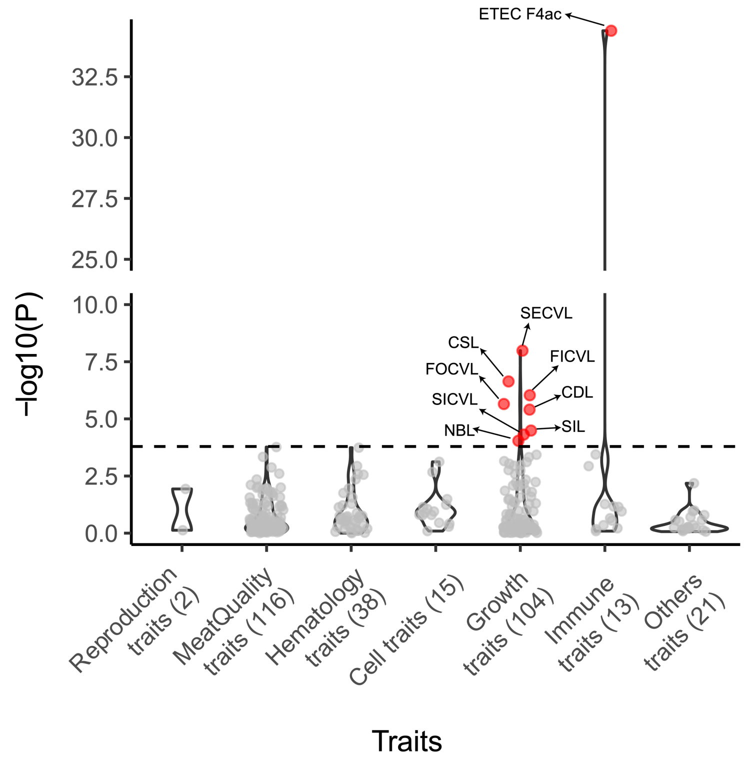

Supplementary Fig. 13 The significance of difference of 309 phenotypes between Hap1 and Hap2 of *MUC13*. Each point represents a phenotype. The y-axis denotes statistical significance tested by Student's test (two-sided) or Pearson's Chi-squared test. Reported log transformed *P* Values are nominal (i.e. not corrected for multiple testing). The numbers in parentheses represent the number of traits. ETEC F4ac, Enterotoxigenic *Escherichia coli* F4ac; CSL, Carcass straight length; CDL, Carcass diagonal length; SIL, Small intestine length; FOCVL, Fourth cervical vertebra length; FICVL, Fifth cervical vertebra length; SICVL, Sixth cervical vertebra length; SECVL, Seventh cervical vertebra length; NBL, total length of cervical vertebra.

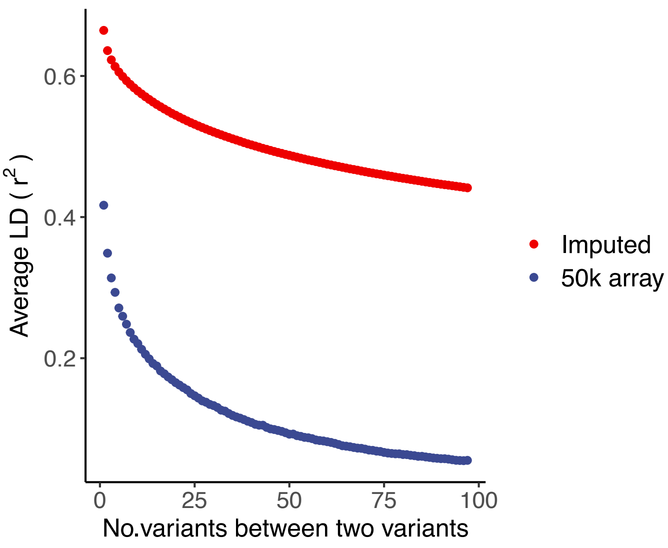

Supplementary Fig. 14 Average Linkage Disequilibrium between all variant pairs. The y-axis denotes the average LD degree measured by r^2. The x-axis denotes the number of variants at chromosomes between two variants. Dots represent average LD under different distances measured by the number of variants between two variants.

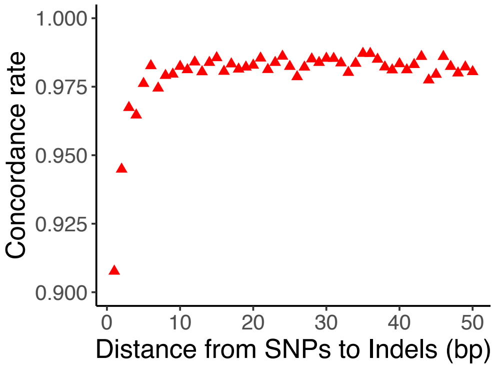

Supplementary Fig. 15 The accuracy of SNPs that are within 1-50 bp of INDELs. Each triangle in red represents a set of SNPs near Indels. The y-axis shows the concordance rate between sequence genotypes and array genotypes.

Supplementary Table 1 Chip-based haplotype analysis for top SNPs at novel loci from imputation GWAS.

Chr	Top SNP	MAF	No.Hap	Begin.Hap	End.Hap	Df	F_value	*P* value
3	3_106129949	0.025	25	105,756,806	106,275,479	24	3.88	6.9E-10
5	5_62861562	0.012	29	62,753,754	63,543,086	28	11.11	9.0E-47
8	8_66234703	0.319	25	65,489,709	66,846,245	24	9.87	2.6E-35
9	9_10302512	0.012	12	10,180,053	10,403,658	11	5.87	1.7E-09
13	13_162125842	0.027	18	160,745,027	162,777,523	17	7.95	6.1E-20

Chr, chromosome; MAF, minor allele frequency; No.Hap, the number of haplotype pattern; Begin.Hap, start position of haplotype; End.Hap, end position of haplotype; Df, degree of freedom; F_value, F value in analysis of variance (ANOVA); *P* value, *P* value in ANOVA. The *P* values are one-sided. Reported *P* values are nominal (i.e. not corrected for multiple testing).

Supplementary Table 2 The frequency of Hap1 of *MUC13* across breeds harboring at least three individuals in populations EUD, NCD, SCD, and Cross. EUD, European domestic pigs; NCD, Northwestern Chinese domestic pigs; SCD, Southeastern Chinese domestic pigs; Cross, European and Eurasian crossbred pigs.

Population	Breed	Sample size	Frequency of Hap1	
EUD	DU	29	0.52	0.67
EUD	PT	6	0.67	
EUD	LW1	62	0.69	
EUD	LW2	5	0.70	
EUD	LR	25	0.74	
EUD	YCT	11	0.86	
NCD	MIN	6	0.33	0.77
NCD	SCT1	50	0.56	
NCD	BAM	6	0.58	
NCD	TT	12	0.67	
NCD	LWU	75	0.97	
NCD	HT	6	1.00	
SCD	LUC	6	0.33	0.44
SCD	BMX	84	0.39	
SCD	EHL	132	0.40	
SCD	SCT2	12	0.42	
SCD	GST	14	0.50	
SCD	JH	6	0.50	
SCD	WZS	6	0.50	
SCD	WA	6	0.67	
SCD	YNT	12	0.79	
SCD	NJ	6	0.92	
Cross	F2	52	0.46	0.51
Cross	F1	44	0.47	
Cross	F3	44	0.47	
Cross	SUT	63	0.48	
Cross	DLY	43	0.52	
Cross	WDU	10	0.65	
Cross	LA	9	0.83	

BAM, Bamei; BAS, Baoshan; BMX, Bamaxiang; DU, Duroc; EHL, Erhualian; F1/F2/F3, Eurasian Crossbred; GST, Gansu Tibetan; HT, Hetao; JH, Jinhua; LA, Lean spotted pig; LR, Landrace; LUC, Luchuan; LW, LargeWhite; LWU, Laiwu; NJ, Neijiang; PT, Pietrain; SCT, Sichuan Tibetan; SUT, Sutai; TT, Tibetan Tibetan; WA, Wanan spotted; WDU, White Duroc; WZS, Wuzhishan; YCT, Yucatan; YNT, Yunnan Tibetan.
