# Supplementary material for: Accurate haplotype construction and detection of selection signatures enabled by high quality pig genome sequences
Source: Nat Commun. 2023 Aug 23;14:5126. doi: 10.1038/s41467-023-40434-3 (PMC10447580; doi:10.1038/s41467-023-40434-3)
Supplement: Supplementary file 3 — Description of Additional Supplementary Files [file 41467_2023_40434_MOESM3_ESM.pdf]

File Name: Supplementary Data 1
Description: Details of re-sequenced samples in terms of breed, abbreviation, group, pedigree, genome coverage, and average depth.

File Name: Supplementary Data 2
Description: Leading variants identified by imputation GWAS for IMF. Wald test is employed to estimate the significance of variants by gemma v0.98 software. The *P* Values are two-sided and nominal (i.e. not corrected for multiple testing).

File Name: Supplementary Data 3
Description: Variants located in the balancing selection haplotype block of *MUC13*.

File Name: Supplementary Data 4
Description: The haplotypes of *MUC13* associate with more than 300 phenotypes (pertaining to reproduction, meat quality, hematology, cell, growth, and immune) in the F2 population. The significances (*P* Value) of phenotypic differences between Hap1 and Hap2 are calculated by Student's t test (two-sided). Reported *P* Values are nominal (i.e. not corrected for multiple testing).

File Name: Supplementary Data 5
Description: Candidate variants under positive selection related to pig stature.
