# Supplementary material for: Accurate haplotype construction and detection of selection signatures enabled by high quality pig genome sequences
Source: Nat Commun. 2023 Aug 23;14:5126. doi: 10.1038/s41467-023-40434-3 (PMC10447580; doi:10.1038/s41467-023-40434-3)
Supplement: Supplementary file 9 — Reporting Summary [file 41467_2023_40434_MOESM9_ESM.pdf]

nature portfolio

NCOMMS-22-27146
Corresponding author(s): Lusheng Huang, Zhiyan Zhang
Last updated by author(s): May 12, 2023

Reporting Summary

Nature Portfolio wishes to improve the reproducibility of the work that we publish. This form provides structure for consistency and transparency in reporting. For further information on Nature Portfolio policies, see our Editorial Policies and the Editorial Policy Checklist.

Statistics

For all statistical analyses, confirm that the following items are present in the figure legend, table legend, main text, or Methods section.

n/a	Confirmed	
☐	☒	The exact sample size (*n*) for each experimental group/condition, given as a discrete number and unit of measurement
☐	☒	A statement on whether measurements were taken from distinct samples or whether the same sample was measured repeatedly
☐	☒	The statistical test(s) used AND whether they are one- or two-sided<br>*Only common tests should be described solely by name; describe more complex techniques in the Methods section.*
☒	☐	A description of all covariates tested
☐	☒	A description of any assumptions or corrections, such as tests of normality and adjustment for multiple comparisons
☐	☒	A full description of the statistical parameters including central tendency (e.g. means) or other basic estimates (e.g. regression coefficient) AND variation (e.g. standard deviation) or associated estimates of uncertainty (e.g. confidence intervals)
☐	☒	For null hypothesis testing, the test statistic (e.g. *F*, *t*, *r*) with confidence intervals, effect sizes, degrees of freedom and *P* value noted<br>*Give P values as exact values whenever suitable.*
☒	☐	For Bayesian analysis, information on the choice of priors and Markov chain Monte Carlo settings
☒	☐	For hierarchical and complex designs, identification of the appropriate level for tests and full reporting of outcomes
☐	☒	Estimates of effect sizes (e.g. Cohen's *d*, Pearson's *r*), indicating how they were calculated

Our web collection on statistics for biologists contains articles on many of the points above.

Software and code

Policy information about availability of computer code

Data collection	Software prefetch v2.9.6 is used to download the 207 resequencing data from the NCBI SRA database.
Data analysis	The codes for variants calling, population structure analyses, construction of the haplotype reference panel, imputation of genotypes, breeding value estimation, balancing selection analyses and positive selection analyses are available from the GitHub repository https://github.com/xinkaitong/1k-pig-genomes<br>The Softwares used are listed in the following:<br>Variants calling: Fastp v0.20.0, BWA v0.7.17, Mosdepth v0.3.2, Picard v2.21.4, GATK v4.1.7.0, bcftools v1.9, Plink v1.9;<br>Population structure analyses: Plink v1.9, PHYLIP v3.69, FigTree v1.4.4;<br>Construction of the haplotype reference panel: HapCUT2 v1.3.1, LINKPHASE3 v1.0, SHAPEIT4 v4.4.2;<br>Imputation of genotypes: IMPUTE5 v1.5.5;<br>Breeding value estimation: gcta v1.93.2Beta;<br>Balancing selection analyses: BetaScan2 v1.0, VCFtools v0.1.13, LDBlockShow v1.40, Clustalo v1.2.4, Fasttree v2.1.11;<br>Positive selection analyses: VCFtools v0.1.13, rehh v3.2.2 package;

For manuscripts utilizing custom algorithms or software that are central to the research but not yet described in published literature, software must be made available to editors and reviewers. We strongly encourage code deposition in a community repository (e.g. GitHub). See the Nature Portfolio guidelines for submitting code & software for further information.

Data

Policy information about availability of data

All manuscripts must include a data availability statement. This statement should provide the following information, where applicable:

- Accession codes, unique identifiers, or web links for publicly available datasets
- A description of any restrictions on data availability
- For clinical datasets or third party data, please ensure that the statement adheres to our policy

The haplotype data, including genotype data and phasing information, generated in this study have been deposited in the National Genomics Data Center database (NGDC) under accession code GVM000479 (https://ngdc.cncb.ac.cn/gvm/getProjectDetail?project=GVM000479). The 740 raw resequencing genome datasets generated in this study have been deposited in NGDC/GSA under accession code CRA011506 (https://ngdc.cncb.ac.cn/gsa). It is available under restricted access for unpublished work involving genome structure variation and evolutionary of pig, access can be obtained by written request to L.H. (lushenghuang@hotmail.com) who will aim to respond to requests within 2 weeks. The 149 genomes we previously published are publicly available 26, 29-32 . The details of all the genomes analyzed in the study, including the 207 publicly available resequencing datasets we retrieved, are included in Supplementary Data 1. The information on the pedigree structure is provided in Supplementary Data 1. The genotypes of individuals and the phenotypic data of IMF from Ding et al. 2 are available at https://figshare.com/s/be3bb2047df324c8a77e. The GWAS summary statistics generated in the study have been deposited in NGDC under accession code GVP000007 (https://bigd.big.ac.cn/gvm/getProjectDetail?Project=GVP000007). The summary statistics for phenotypic associations of gene MUC13 are provided in Supplementary Data 4.

Human research participants

Policy information about studies involving human research participants and Sex and Gender in Research.

Reporting on sex and gender	N/A
Population characteristics	N/A
Recruitment	N/A
Ethics oversight	N/A

Note that full information on the approval of the study protocol must also be provided in the manuscript.

Field-specific reporting

Please select the one below that is the best fit for your research. If you are not sure, read the appropriate sections before making your selection.

☒ Life sciences ☐ Behavioural & social sciences ☐ Ecological, evolutionary & environmental sciences

For a reference copy of the document with all sections, see nature.com/documents/nr-reporting-summary-flat.pdf

Life sciences study design

All studies must disclose on these points even when the disclosure is negative.

Sample size	There is no sample size calculation being performed. As a discovery project, the main purpose of this study is to construct pig haplotype reference panel and detect selection signature. The more sample we collect, the more resequencing data we could use to construct haplotypes, which enable accurate construction. We try our best to sequence 740 pigs and reuse 149 of our previously published pig genomes. Besides, as much as possible, we retrieved 207 pig genome resequencing data from the NCBI SRA database. Generally, ~1k pigs could be used to calculate linkage disequilibrium for haplotype construction.
Data exclusions	We removed reads with ≥ 10% missing ("N") bases or quality score ≤ 20 for ≥ 50% of bases, which could reduce low-quality reads with high error rate. Because high IBS (Identity by state) between sample pairs indicates the repeated sample, we exclude duplicated samples with IBS > 0.95 between each sample pair, resulting in a removal of 61 duplicated individuals.
Replication	As a discovery project, the main purpose of this study is to construct pig haplotype reference panel and detect selection signature. Replication was performed by repeating selection-signature detection in different pig groups. Four replications were conducted in balancing selection, and all findings were successfully repeated.
Randomization	As a discovery project, the main purpose of this study is to construct a pig haplotype reference panel and detect selection signature using maximal sample size as much as possible. In haplotypes construction, all individuals were included in the analyses. In balancing selection detection, all individuals from Southeastern Chinese domestic pigs, Northwestern Chinese domestic pigs, Crossbred pigs, and European domestic pigs were used for analysis. In positive selection detection, all individuals from two large stature pigs and two small stature pigs were used for identifying loci under selection. So this is not relevant to this study.
Blinding	As a discovery project, the main purpose of this study is to construct a pig haplotype reference panel and detect selection signature using

Blinding	maximal sample size as much as possible. In haplotypes construction, all individuals were included in the analyses. In balancing selection detection, all individuals from Southeastern Chinese domestic pigs, Northwestern Chinese domestic pigs, Crossbred pigs, and European domestic pigs were used for analysis. In positive selection detection, all individuals from two large stature pigs and two small stature pigs were used for identifying loci under selection. So this is not relevant to this study.

Reporting for specific materials, systems and methods

We require information from authors about some types of materials, experimental systems and methods used in many studies. Here, indicate whether each material, system or method listed is relevant to your study. If you are not sure if a list item applies to your research, read the appropriate section before selecting a response.

Materials & experimental systems

n/a	Involved in the study
☒	☐ Antibodies
☒	☐ Eukaryotic cell lines
☒	☐ Palaeontology and archaeology
☐	☒ Animals and other organisms
☒	☐ Clinical data
☒	☐ Dual use research of concern

Methods

n/a	Involved in the study
☒	☐ ChIP-seq
☒	☐ Flow cytometry
☒	☐ MRI-based neuroimaging

Animals and other research organisms

Policy information about studies involving animals; ARRIVE guidelines recommended for reporting animal research, and Sex and Gender in Research

Laboratory animals	The F2 pig population used for phenotypic analyses were slaughtered for recording related traits at day 240±3.
Wild animals	We captured 66 wild boars from mountains using tranquilizer guns and collected their ear tissues immediately. The exact ages of all wild boars were unknown. In order to collect samples from other locations, all wild boars were anesthetized and transported to the laboratory. The project was supported by the government and Animal Care and Use Committee (ACUC) in Jiangxi Agricultural University. The ethics committee of Jiangxi Agricultural University specifically approved this study.
Reporting on sex	The main purpose of this study is to construct a pig haplotype reference panel and detect selection signature. Only autosomal variants were used for the construction of haplotype reference panel and selection-signature detection. Sex has little influence on final results. So, we just imputed their sex by summarizing the number of variants on Y-chromosome.
Field-collected samples	All experimental pigs were sampled under standard procedures within 30 min at room temperature.
Ethics oversight	The ethics committee of Jiangxi Agricultural University specifically approved this study. All animal work was conducted according to the guidelines for the care and use of experimental animals established by the Ministry of Agriculture of China. The project was also approved by Animal Care and Use Committee (ACUC) in Jiangxi Agricultural University.

Note that full information on the approval of the study protocol must also be provided in the manuscript.
